# Atypical Presentations in Melioidosis: A Case-Based Review from Endemic Regions

**DOI:** 10.3390/idr18010015

**Published:** 2026-02-03

**Authors:** Saurav Jyoti Patgiri, Anukalpa Saikia, Sushmita Yadav, Md. Atique Ahmed, Luna Adhikari, Chimanjita Phukan, Chiranjay Mukhopadhyay, Harpreet Kaur

**Affiliations:** 1 ICMR Regional Medical Research Centre North East, Dibrugarh 786001, India; anukalpasaikia77@gmail.com (A.S.); sushmitayadav70@gmail.com (S.Y.); ahmed.atique@icmr.gov.in (M.A.A.); 2 Department of Microbiology, Sikkim Manipal Institute of Medical Sciences, Gangtok 737102, India; luna.a@smims.smu.edu.in; 3 Department of Microbiology, Assam Medical College and Hospital, Dibrugarh 786002, India; chimanjitaphukan@gmail.com; 4 Department of Microbiology, Kasturba Medical College, Manipal 576104, India; chiranjay.m@manipal.edu; 5 Communicable Diseases, ICMR Headquarters, Ansari Nagar, New Delhi 110029, India; kaurh.hq@icmr.gov.in

**Keywords:** melioidosis, *Burkholderia pseudomallei*, atypical infections, abscess, neuro-melioidosis, imported infections, tropical medicine

## Abstract

Background: Melioidosis, caused by *Burkholderia pseudomallei*, is a severe and often underdiagnosed infection endemic to South Asia, Southeast Asia, and northern Australia. While pneumonia and sepsis are the classical presentations, the disease is increasingly recognized for its diverse and atypical clinical manifestations. Objective: The objective is to improve diagnostic accuracy and increase clinical awareness in both endemic and non-endemic settings by reviewing and classifying atypical presentations of melioidosis that have been documented in the literature. Methods: A narrative, case-based review was conducted using 238 published case reports and series from endemic and transitional regions during the period from 2000 to 2025. Cases with non-respiratory presentations or anatomical locations not commonly linked to melioidosis were classified as atypical. Clinical syndromes were used to classify the extracted cases, and common patterns in presentation, diagnosis, and outcome were examined. Results: One hundred and sixty published articles were included after a full text review. The most frequent atypical presentations included neurological involvement (e.g., brain abscess, encephalomyelitis), musculoskeletal infections (osteomyelitis, myositis), thyroid abscess, tubo-ovarian abscess, and dermatologic manifestations such as erythema nodosum. Imported and pediatric cases were also found. Numerous cases were misidentified as cancer, fungal infections, or tuberculosis. Among risk factors, diabetes mellitus was the most prevalent. Non-specific symptoms, a lack of laboratory capacity, and incorrect pathogen identification frequently resulted in delays in diagnosis. Conclusions: In endemic areas, melioidosis should be taken into account when making a differential diagnosis of a variety of clinical syndromes, especially in patients who have diabetes or have had relevant environmental exposure. Poor outcomes and diagnostic delays are greatly exacerbated by atypical presentations. Improving diagnostic capabilities and raising awareness are crucial to lessening the worldwide burden of this often ignored but potentially deadly infection.

## 1. Introduction

Melioidosis, caused by the environmental Gram-negative bacillus *Burkholderia pseudomallei*, is a potentially life-threatening infectious disease endemic to Southeast Asia, South Asia, and northern Australia [1,2]. Traditionally associated with pneumonia and sepsis in immunocompromised individuals, particularly those with diabetes mellitus, melioidosis is recognized to involve almost all systems and present with protean clinical manifestations [3,4]. Recent years have seen a rising number of atypical presentations across multiple organ systems, often leading to misdiagnosis and delayed treatment [5,6,7].

The organism resides in soil and surface water, with infection typically acquired through percutaneous inoculation, inhalation, or, less commonly, ingestion [8,9]. Heavy rainfall, flooding, and occupational exposure (e.g., farming, construction work) significantly increase the risk of disease acquisition [9,10]. Diabetes mellitus is a well-documented predisposing factor for melioidosis, with some studies reporting three times higher incidence of melioidosis in diabetics [11]. While bacteremic pneumonia remains the most commonly reported form, *B. pseudomallei* has been documented to cause abscesses and lesions in virtually every organ system, including the brain, bone, thyroid, genitourinary tract, and skin [4,5,8,12,13]. This wide spectrum of disease presentation is further compounded by its ability to remain latent for years, reactivating under immunosuppressive conditions [3].

Diagnosing melioidosis remains a challenge in resource-limited settings. It is frequently mistaken for tuberculosis, fungal infections, or malignancies, especially when it presents with mediastinal lymphadenopathy, chronic cutaneous lesions, or musculoskeletal complaints [2,6,7,9]. Coinfections with other bacterial agents is not uncommon and is often associated with higher mortality rates [14]. In many cases, blood cultures are sterile, and serological testing may be negative despite active infection [12]. These diagnostic hurdles are particularly concerning in resource-limited settings, where laboratory capabilities are often insufficient for the accurate identification of *B. pseudomallei* [2]. Automated and molecular methods have significantly higher sensitivity and specificity when compared to conventional methods for diagnosis of melioidosis [15].

Despite being underrecognized, the global burden of melioidosis is substantial, with an estimated 165,000 cases and 89,000 deaths annually [16]. Recent data from South Asia suggest an expanding epidemiological footprint, with increasing reports of locally acquired cases from regions once considered non-endemic, such as Nepal and North India [7,17]. Imported cases among travelers and migrant workers further underscore the global relevance of the disease [3].

This article aims to highlight the atypical and under-reported manifestations of melioidosis through a case-based review, drawing from the published literature in endemic and emerging regions. By categorizing presentations across neurological, musculoskeletal, visceral, dermatologic, and urinogenital systems, we hope to enhance clinician awareness and advocate for the inclusion of melioidosis in the differential diagnosis of diverse clinical syndromes, particularly in patients with relevant exposure history and risk factors.

## 2. Materials and Methods

This study is a case-based, narrative review of the literature that focuses on unusual clinical manifestations in melioidosis. We included case reports, series, and observational studies that described melioidosis manifesting outside the classical pulmonary or septicaemic forms.

A literature search was carried out on PubMed using the search words “(Melioidosis) and (Case Report)”. Eligibility criteria included English-language articles published between 2000 and 2025, with confirmed microbiological or serological evidence of *B. pseudomallei*. Only those articles where full text was available publicly were selected. Reports of melioidosis in animals were excluded and so were preprints. Atypical presentations were defined as those involving organ systems not commonly affected or cases with misleading clinical signs. For the purpose of this analysis, atypical melioidosis was considered as all those cases of melioidosis where pulmonary and septicaemic forms of melioidosis were absent and other systems were involved.

Data were extracted from 238 uploaded case reports and case series spanning regions such as India, Sri Lanka, Thailand, Australia, Nepal, China, and Japan. Cases were grouped by organ system and analyzed descriptively.

## 3. Results

A total of one hundred and sixty articles met the eligibility criteria and were included after screening. Clinical manifestations were categorized according to the primary organ system involved. Broadly, the following classifications were made based on the systems involved and few additional categories were added based on the case reports screened (Table 1).

### 3.1. Neurological Involvement

Neuro-melioidosis is an uncommon but severe manifestation of melioidosis caused by *Burkholderia pseudomallei*, characterized by central nervous system involvement. The condition mimics various neurological syndromes including encephalomyelitis, brain abscess, transverse myelitis, and Guillain–Barre syndrome. Neurological involvement, though rare (3% to 10% of all melioidosis cases), can result in devastating complications [18]. Neuro-melioidosis is often misdiagnosed due to its protean manifestations and overlaps with other neurological disorders. Increasing case reports from endemic and non-endemic areas underscore the need for heightened clinical awareness.

#### 3.1.1. Presentation

Neuro-meliodosis can present as a very wide spectrum. The most common presentation is encephalomyelitis, characterized by fever, altered sensorium, and motor deficits [19,20,21,22]. However, encephalomyelitis may not be the first presentation in a case of melioidosis. It may develop later, subsequent to a primary focus elsewhere, as demonstrated in the case of an 11-year-old girl from Chennai, India, who developed acute disseminated encephalomyelitis following a history of left-gluteal abscess which was treated with surgical drainage [19]. Clinical suspicion coupled with serial brain imaging and brain biopsy helped in establishing the diagnosis of neuro-melioidosis [21]. Motor deficits like sudden onset of bilateral lower limb weakness, numbness, and urinary retention are also possible as initial presentations in a case of neuro-melioidosis [21]. Longitudinally extensive transverse myelitis (LETM) characterized by paraparesis and quadriparesis has also been documented in two cases from Sri Lanka [23]. Another common presentation of neuro-melioidosis is brain abscess, often visualized as a ring-enhancing lesion, which is commonly misdiagnosed as tuberculosis or toxoplasmosis [18]. Cranial nerve palsies involving the facial nerve are also common in neuro-melioidosis [24]. Involvement of the vagus nerve has also been reported in a case of pulmonary melioidosis [25]. Hoarseness, caused by the involvement of the recurrent laryngeal nerve, is an indirect indicator of vagus nerve neuritis in melioidosis, as shown in a case report from Australia [25]. Nerve involvement in neuro-melioidosis is usually secondary, with the primary focus being located in the brain or the spine. Rhombencephalitis or brainstem encephalitis is another uncommon neurological presentation of melioidosis. Multiple asymmetrical cranial nerve palsies, cerebellar dysfunction, sensory loss, and seizures may be the initial presentation in rare cases [26]. Optic neuritis, characterized by acute, painless loss of vision in one eye, was the presenting complaint of a female teacher with no pre-existing comorbidities [26]. Melioidosis was confirmed in both these cases of rhombencephalitis and optic neuritis after positive cultures were obtained from CSF/blood samples [26]. Guillain–Barre syndrome is very rarely the initial clinical presentation of melioidosis. It is usually a complication arising out of this disease and should be suspected in melioidosis cases involving lower limb weakness [27,28]. Treatment options include intravenous immunoglobulins and plasmapheresis; however, in cases where GBS is detected during active melioidosis, plasmapheresis should be used preferentially since immunoglobulins have an immunomodulatory role, which may complicate melioidosis recovery [27]. Meningitis coupled with scalp abscess has also been reported in Karnataka, India, in a male farmer with diabetes [26]. Inadequate therapy in melioidotic mycotic aneurysm can also lead to relapses presenting as acute meningitis [29].

#### 3.1.2. Diagnosis

Diagnosing a case of neuro-melioidosis requires a high degree of suspicion and the availability of a well-equipped laboratory and access to imaging facilities. Clinical features and radiological imaging can mimic a number of other conditions, especially in areas which are non-endemic for melioidosis [30]. CSF cultures are also frequently sterile. Laboratory investigations like CSF findings can be inconclusive. Elevated CSF proteins without a significant drop in glucose levels with mononuclear cells may suggest neuro-melioidosis [23]. MRI seems to be the best imaging modality for diagnosis. Localized lesions in the brain such as abscesses and micro-abscesses are visible as hyperintense ring-enhancing lesions. T2-weighted hyperintensity in the cortico-spinal tracts and thickening of the involved nerves are salient MRI findings in neuro-melioidosis [23,25]. Brain biopsy proved to be better than CSF for isolating *B. pseudomallei* in neuro-melioidosis cases [19,20,31].

#### 3.1.3. Treatment and Recovery

Treatment of neuro-melioidosis is similar to the treatment of melioidosis per se, with additional interventions adopted depending on the clinical presentation. Intravenous meropenem/imipenem was the mainstay of treatment during the initial intensive phase followed by trimethoprim/sulfamethoxazole orally during the continuation phase. Intravenous ceftazidime was also used with success instead of imipenem/meropenem in a number of neuro-melioidosis cases included in the current review [18,21,25,31,32,33,34,35,36]. Cases with brain abscess frequently required craniotomy and abscess drainage [37,38]. Patients with GBS-like symptoms were given intravenous immunoglobulins; plasmapheresis was also used successfully in one case [27,28,32]. Recovery in neuro-melioidosis cases was found to be mixed, with some patients showing full recovery, whereas some recovered with significant disabilities. The mortality rate was surprisingly not very high, with four recorded deaths in the 24 case studies screened [22,26,33,36]. Patients with risk factors and presenting with septicaemia had worse outcomes [33]. Almost 50% of the cases had some amount of neurological deficits like persistent paraplegia, hoarseness, quadriplegia, and so on [21,25,34]. Initial time to diagnosis, prevalence of risk factors, and proper antibiotic therapy were the main factors driving patient recovery.

### 3.2. Musculoskeletal Involvement

Osteomyelitis, pyomyositis, spinal infections, and septic arthritis are among the many musculoskeletal manifestations of melioidosis. These disorders can manifest separately or together, and because of their diverse clinical presentations that can resemble those of other common infections or inflammatory diseases, they frequently present diagnostic difficulties.

In more than half of the documented cases, diabetes mellitus is the most prevalent comorbidity linked to musculoskeletal melioidosis [39,40,41,42,43,44]. Additional risk factors include immunosuppression, heavy drinking, thalassemia, chronic kidney disease, and exposure to soil or standing water at work [43,44,45,46,47,48]. There have been some reports from non-endemic areas with no clear travel history, suggesting potential for underrecognized endemicity or latency [49].

#### 3.2.1. Soft Tissue Involvement

Musculoskeletal melioidosis can present with a wide clinical spectrum. Myositis and soft tissue involvement is the most common manifestation. Although infective myositis is uncommon, it may occur in diabetic patients with no antecedent trauma, and is often mistaken for cellulitis or necrotizing fasciitis [39,50]. Two Sri Lankan cases highlight rapid progression to myonecrosis, requiring urgent surgical intervention [39]. Initial imaging may suggest focal myositis without abscess, leading to diagnostic delays. Culture from muscle biopsy or pus is often definitive [50]. Treatment with intravenous meropenem and ceftazidime showed good recovery in both cases [39,50]. A case of pyomyositis presenting as a suprapubic mass was also reported from Malaysia and responded well to ceftazidime and oral co-trimoxazole [47].

In addition, musculoskeletal melioidosis may mimic tubercular infection, especially in endemic regions. A diabetic from Karnataka, India, presented with an abscess in the left loin which resembled a tubercular cold abscess on radiography [51]. However, pus from the abscess grew *B. pseudomallei* and the patient responded well to ceftazidime and co-trimoxazole. There are reports from Chennai, India, where a type 2 diabetic farmer presented with paraspinal abscess and was initially misdiagnosed as a case of tubercular vertebral osteitis [52]. Another case of spondylodiscitis accompanied by anterior epidural and prevertebral collections showed radiological findings consistent with tuberculous etiology, but was later confirmed as melioidosis by a lateral flow assay [53]. Scalp abscess with involvement of the parietal bone and intracranial infiltration in immunocompromised cases like HIV sero-positives and diabetics should also raise the suspicion of melioidosis [54,55]. These cases with intracranial involvement can often be fatal [55].

A case of bloodstream infection with paravertebral abscess caused by *B. pseudomallei* was reported from China, and the source of infection was difficult to assess initially. Detailed medical history revealed that the patient had undergone lumbar acupuncture treatment a week before the symptoms. The infection had probably spread from the procedure leading to an initial paravertebral abscess and then a bloodstream infection [56]. An American teenager who had traveled to northeast Thailand developed multiple thigh abscesses at the site of mosquito bites; cultures yielded *B. pseudomallei* and the patient recovered fully after appropriate therapy with ceftazidime and doxycycline [57]. The use of positron emission tomography–computed tomography (PET-CT) for diagnosis of melioidosis involving the humerus in a traveler from Cambodia has also been documented [58]. Atypical presentations in healthy individuals with a positive travel history to endemic sites should also raise the suspicion of melioidosis.

#### 3.2.2. Joint Involvement

Large joints like the ankle, knee, or hip are commonly affected by *B. pseudomallei* and present as septic arthritis [43]. The necessity of early detection and vigorous treatment is highlighted by two fatal cases of ankle arthritis from Assam, India [43]. Both these patients were diabetics and despite adequate therapy, they succumbed to the infection. On the other hand, cases treated with joint washout and ongoing antibiotic therapy have shown positive results [42].

Chronic granulomatous osteomyelitis is an insidious form often confused with tuberculosis or other bacterial causes. Atypical sensitivity profiles of *B. pseudomallei* may result in treatment failure unless cultures are obtained [59]. One case from India documented resistance to ceftazidime, requiring ciprofloxacin and amoxicillin–clavulanate [59]. Osteomyelitis due to *B. pseudomallei* has been reported frequently in the parietal bone and femur, with most cases showing good recovery rates with intravenous ceftazidime or meropenem [48,60,61,62,63,64,65].

Spinal melioidosis is rare but serious. Cases include intraosseous abscesses of the sacrum, paraspinal muscle involvement with paraplegia, and vertebral osteomyelitis [44,66]. Diagnosis often requires MRI and biopsy confirmation. Prolonged intravenous therapy with carbapenems or ceftazidime, followed by oral eradication therapy, is critical. Both the cases showed good recovery.

A rare case of peri-prosthetic infection involving an old medial malleolar screw highlights the potential for latency and reactivation [41]. Resolution was achieved through the use of culture-guided therapy in conjunction with surgical debridement and screw removal. Involvement of prosthetic material in melioidosis is rare, with only few reports available in the literature. There were two reports of involvement of prosthetic valve leading to endocarditis and cardiac pacemaker involvement reported previously [67,68]. An uncommon case involving the temporomandibular joint and masticator muscles was reported from India; it was the first of its kind to present with intracranial extension and a locked jaw [40]. In a different case, an 18-year-old rice farmer had recurring splenic micro-abscesses and buttock abscesses, which yielded *B. pseudomallei* on culture from pus samples. However, the patient had normal blood glucose and creatinine levels and responded well to intravenous ceftazidime therapy. A possible source of infection was from the paddy field [69]. 

### 3.3. Cutaneous/Skin Involvement

The clinical manifestations of cutaneous melioidosis are diverse and frequently reflect the patient’s immune status and the route of inoculation. Understanding the variety of skin manifestations is essential for effective treatment.

#### 3.3.1. Ulcerative Lesions

The most frequently reported types of cutaneous melioidosis are ulcerative lesions. According to one report, a 26-year-old man who had been traveling extensively in Asia and had been bitten by an insect in Thailand had a persistent distal leg ulcer. Multiple courses of oral antibiotics were ineffective in treating the ulcers, demonstrating the recalcitrance of melioidosis ulcers. [70]. Although initially identified as *B. thailandensis*, the antibiotic profile closely resembled that of *B. pseudomallei*. In a different case, a 90-year-old woman who visited Bangladesh developed an ulcerated and erythematous cutaneous lesion on her left elbow that did not heal. Despite topical antimicrobial treatment, the lesion worsened, highlighting the possibility that melioidosis could manifest in elderly travelers [71]. An Israeli traveler returning from Thailand also presented with skin lesions [72]. He did not have any risk factors for the disease and responded well to 12 weeks of oral antibiotic therapy with trimethoprim–sulfamethoxazole.

#### 3.3.2. Abscess

Cutaneous melioidosis can also present as an abscess. A 26-year-old man in the UK was the subject of a case report that described a cutaneous thigh abscess caused by melioidosis. The patient was of Chinese origin and had a positive travel history to his native country. This case illustrated that melioidosis, although rare in the UK, should be considered in the differential diagnosis of soft tissue abscesses, even in non-endemic areas [73].

#### 3.3.3. Cellulitis

Cellulitis is another cutaneous manifestation of melioidosis, though it is not very common. Venous thrombosis and thrombophlebitis complicated cellulitis in the left lower limb of a 48-year-old male Bangladeshi with diabetes, according to one report. As demonstrated by this case, melioidosis can manifest as a severe soft tissue infection and can be linked to complications such as thrombosis, especially in those with risk factors [74].

#### 3.3.4. Rare Inflammatory Conditions

Apart from ulceration, abscess and cellulitis, a number of other atypical presentations have also been reported in the literature. In Thailand, a 32-year-old woman with chronic myeloid leukemia showed signs of *B. pseudomallei*-induced infective panniculitis on her left forearm. Her medical history revealed that she was involved in cultivating cacti as a hobby and had repeated exposures to cactus pricks on her upper extremities. This is a rare manifestation of cutaneous melioidosis, mimicking other inflammatory conditions of the subcutaneous fat [75]. A 53-year-old woman from Sri Lankan developed Sweet syndrome characterized by fever and painful skin lesions, secondary to melioidosis. Histopathological examination of skin lesions over the face and forearm showed predominantly neutrophilic infiltrates consistent with Sweet syndrome and panniculitis. This unusual association broadens the spectrum of cutaneous manifestations of melioidosis [76].

#### 3.3.5. Erythema Nodosum

Erythema nodosum is another uncommon presentation of cutaneous melioidosis. Two children in Australia presented with lower limb erythema nodosum. High titres of the indirect hemagglutination assay for melioidosis were used to make the diagnosis. This report raises the possibility of an unreported link between erythema nodosum and melioidosis [77]. An erythema nodosum-related severe haemorrhagic bleb in a Thai child was also attributed to melioidosis by positive serology. After receiving 12 weeks of therapy for melioidosis, the antibody titre decreased significantly. The possibility of severe cutaneous involvement in melioidosis, particularly in endemic areas, is demonstrated by this case [5]. In Malaysia, erythema nodosum was observed on both upper forearms, anterior thighs, and the lower back of a 39-year-old woman with type II diabetes mellitus. *Burkholderia pseudomallei* was found in blood cultures, confirming melioidosis. The histological results from the subcutaneous nodules were consistent with erythema nodosum [78].

#### 3.3.6. Necrotizing Fasciitis

A case of cutaneous melioidosis developing into necrotizing fasciitis was described in one report, highlighting the possibility of severe and quick tissue destruction. The patient had a history of psoriasis and alcoholic liver cirrhosis, and worked as a dishwasher at a restaurant. Initially, he developed necrotizing fasciitis over his left ankle but was readmitted after several months with prostatic abscess and then again with an abscess in the left temporal region. Cultures were positive for *B. pseudomallei* for each of the three occasions [79].

#### 3.3.7. Other Cutaneous Lesions

A report from Australia described a soccer player who developed cutaneous melioidosis following an abrasion incident in a soccer field around two and a half months prior. *B. pseudomallei* cultured from the player was identical to that recovered from soil at the injury site by MLST typing, suggesting that melioidosis can result from soil contamination of wounds during sporting activities, even in healthy subjects [80].

#### 3.3.8. Latent Infections

Latency in melioidosis was also documented in a case report describing a man who was taken as a prisoner of war by the Japanese during World War II. He presented with a nonhealing ulcer on his right hand 62 years after the initial exposure [81]. A case of cutaneous melioidosis in France was reported in an adolescent returning from Guadeloupe, highlighting that the disease can occur in unexpected locations [82]. A case report describes a patient with Porphyria cutanea tarda developing secondary to treatment for pulmonary melioidosis with doxycycline and amoxycillin. Cessation of these drugs prescribed during the maintenance phase of melioidosis led to remission of the skin lesions [83]. Successful treatment of localized cutaneous melioidosis with oral antibiotic therapy alone has also been reported from Australia, where a 65-year-old man showed complete recovery after three months of oral trimethoprim/sulfamethoxazole [84]. Similarly, a Danish man who had acquired the infection during a car accident in Malaysia also recovered well after oral therapy [85]. Primary cutaneous melioidosis acquired in Nepal was also reported [86].

From minor skin lesions to serious infections, cutaneous melioidosis can present with a wide range of clinical manifestations. Particularly when dealing with patients who have pertinent exposure and travel histories, clinicians need to keep a high index of suspicion. A reassuring factor in cases of cutaneous melioidosis is that fatalities are comparatively lower and appropriate oral antibiotic therapy alone was sufficient for almost total recovery in a number of cases [84,85].

### 3.4. Visceral Melioidosis

Involvement of visceral organs due to melioidosis is not very common, but a number of case reports have documented this, especially in endemic regions. The liver, spleen, pancreas, kidneys, and peritoneum can all be affected by visceral melioidosis. Prolonged fever, rigors, anorexia, weight loss, and abdominal pain are typical symptoms. Hepatosplenomegaly or sepsis symptoms may also be present in certain patients. In cases of hepatic involvement, distinct imaging indicators like “honeycomb” or “necklace” abscess patterns have been documented. Splenic abscesses can happen alone or in conjunction with pneumonia. Multiple intra-abdominal abscesses are the usual presentation of pancreatic involvement. Vascular problems like aortic pseudoaneurysm or peritonitis from infections linked to dialysis are infrequently reported.

#### 3.4.1. Splenic Abscess

Out of all cases of visceral melioidosis, the most common presentation was that of splenic abscess, sometimes multiple. A case series from Kapit, Malaysia, reported as many as 39 instances of splenic abscess due to *B. pseudomallei* over a span of two years. All cases, except one, recovered with conservative therapy for melioidosis. The only fatality was due to the development of septicaemia and multi-organ failure [87]. Another case from Malaysia also reported multiple splenic micro-abscesses in a 62-year-old diabetic male who later developed necrotizing pneumonia and septicaemic shock. He later succumbed to severe bacteremic melioidosis despite receiving appropriate therapy [88]. Surgical intervention is sometimes required in cases of isolated splenic abscess that do not respond to conservative therapy [89]. Repeated testing may also prove fruitful in non-responders, especially in setups where automated culture systems are used [90]. Although diabetes is a recognized risk factor for melioidosis, those on insulin therapy may be more prone to infection. An 18-year-old Type 1 DM from West Bengal, India, had an isolated splenic abscess which was later confirmed as being caused by *B. pseudomallei*. He was on insulin, and the probable source of infection was identified as the subcutaneous injection site which was probably contaminated during activities like cattle grazing and bathing in ponds [91]. Cases of splenomegaly with granulomatous lesions and the absence of abscess have also been reported to have been caused by *B. pseudomallei* [92]. A case of splenic granuloma was reported as an incidental finding in a 54-year-old man who was treated following a road-traffic accident. He later developed full-blown melioidosis and recovered after treatment [93]. An imported case of melioidosis presenting with splenomegaly and splenic abscess was reported from China in a returning traveler from Laos [94].

#### 3.4.2. Hepatic Involvement

Liver abscess has also been reported in case of visceral melioidosis and may occur in isolation or combined with splenic abscess. Asymmetric loculations of varying size (honeycomb sign) or arranged radially around a dense focus (necklace sign) may be present in some cases [95]. Pyogenic liver abscess in melioidosis-endemic areas and in patients with risk factors should always raise the suspicion of melioidosis, otherwise a diagnosis will be missed or delayed [96].

#### 3.4.3. Pancreatic Abscess

Pancreatic involvement in melioidosis is rare. A report from Brunei reported a 6.2% prevalence of pancreatic involvement in melioidosis cases diagnosed over a five-year period. Radiological investigation showed predominantly multiple micro-abscesses. In all these cases, the pancreas was not involved in isolation; simultaneous involvement of the spleen, liver, psoas muscle, and bloodstream were present. However, all cases responded favorably to conservative treatment without requiring drainage [97].

#### 3.4.4. Peritoneal Involvement

Peritonitis caused by B. pseudomallei is an equally rare phenomenon, with only two reports available in the literature [98]. In a case from Malaysia, a 47-year-old lorry driver, with chronic kidney disease and diabetes, on continuous ambulatory peritoneal dialysis (CAPD) developed peritonitis. B. pseudomallei was isolated from the peritoneal fluid culture and the patient responded well to conservative treatment [98]. An unusual case of mesenteric abscess with peritonitis due to B. pseudomallei was also reported from Thailand [99].

### 3.5. Vascular Involvement

Vascular involvement in melioidosis is a rare but life-threatening manifestation of *Burkholderia pseudomallei* infection. In endemic areas and among returning tourists, mycotic aneurysms, especially those of the aorta and iliac arteries, have become more common. Improving results requires prompt diagnosis, vigorous antibiotic treatment, and suitable vascular surgery. Mycotic aneurysms due to *B. pseudomallei* are estimated to occur in 0.4–7.5% of patients with melioidosis [100]. Risk factors are similar to other forms of melioidosis. Cases have also been reported in immunocompetent travelers returning from endemic regions. The clinical presentation is non-specific and often includes fever, abdominal or back pain, weight loss, and constitutional symptoms. The abdominal aorta, iliac arteries, and, less frequently, the thoracic aorta, are the most commonly affected by mycotic aneurysms [101,102]. Some cases recur after a latent infection or mimic cancer [103].

A case series from China (Hainan Province) reported eight cases of mycotic aneurysm, of which six had abdominal and one each had iliac and mesenteric aneurysms. All had positive cultures and risk factors like hypertension and environmental exposure. These cases were managed with surgery and appropriate antibiotics; however, mortality was high at 25% [100]. A similar series from tropical Australia reported a 3.4% incidence of mycotic aneurysms in 233 melioidosis cases over a five-year period. ICU admission was required for 75% of the cases, and the mortality rate was 25% despite high-resource settings [102]. Aneurysms involving the abdominal aorta and femoral arteries were also reported in three cases from Malaysia. Pseudoaneurysm of the infra-renal part of the abdominal aorta secondary to melioidosis was reported in three cases from Singapore. This is an extremely rare condition and equally difficult to diagnose and treat. All three cases were managed with long-term ceftazidime and surgical intervention involving removal of the infected portion of the aneurysm, followed by reconstruction [104].

Vascular involvement in melioidosis has been managed successfully with surgery and antibiotics (ceftazidime and co-trimoxazole), and complete recovery was achieved in all three cases [101]. However, in a few cases, involvement of the abdominal aorta requires resection and repair by autologous deep vein grafts [105]. A report from France showed mycotic aneurysm involving the left common iliac artery caused by *B. pseudomallei*; the patient responded favorably to imipenem and trimethoprim/sulfamethoxazole therapy [106]. Involvement of the left iliac artery was also reported from Taiwan. However, it was a late presentation, secondary to pulmonary melioidosis, and required a bypass graft. The patient responded well to ceftazidime and had no relapse at eight months’ follow-up [103].

### 3.6. Genitourinary Tract Involvement

Involvement of the genitourinary system in melioidosis is not common but should be suspected in returning travelers and patients from endemic regions. From tubo-ovarian abscesses to prostatic and renal complications, genitourinary melioidosis can manifest with a broad range of clinical syndromes.

#### 3.6.1. Involvement of Female Genitourinary Tract

Tubo-ovarian abscess presents as abdominal pain and diagnosis is difficult without radio-imaging. A report from Thailand documented a case of tubo-ovarian abscess with multiple hepatosplenic abscesses. After left salpingo-oophorectomy and pus drainage, the diagnosis of melioidosis was established by pus culture. The patient responded to intravenous ceftazidime followed by oral co-trimoxazole [8]. A case of acute chorioamnionitis was also reported in an Australian woman who subsequently delivered pre-term. Both mother and child were put on meropenem and recovered with no recurrences [107].

#### 3.6.2. Involvement of Male Genitourinary Tract

Prostatic abscess is another uncommon manifestation of melioidosis. In Norway, two cases of melioidosis with prostatic and splenic abscess were reported. Both the cases had a history of travel to endemic regions like Sri Lanka and Thailand and presented as urinary tract infections [108]. From India, a 64-year-old male was diagnosed with acute right pyelonephritis and prostatic abscess caused by *B. pseudomallei*. Treatment required surgical intervention, but the patient recovered completely [109]. Suppurative epididymo-orchitis and chronic prostatitis has also been reported from France in a 52-year-old male originally from Gabon [110]. Especially in diabetics from endemic areas, the possibility of prostatic abscess being caused by *B. pseudomallei* should be considered [111].

Isolation of *B. pseudomallei* from urine samples is rare, and considering that most urine samples are not handled in a BSL3 facility, the risk of exposure for laboratory staff is limited. Nevertheless, there are reports from France and the U.S. where laboratory staff were exposed to infected urine samples during initial processing for cultures [112,113].

### 3.7. Thoracic/Mediastinal Involvement

Although less frequently reported, thoracic and mediastinal involvement can manifest as vascular complications, pneumonia, lung abscesses, empyema, pericarditis, mediastinal lymphadenitis, and even cardiac infections like endocarditis. Due to its varied radiological features and mimicry of tuberculosis or malignancy, thoracic involvement in melioidosis often presents a diagnostic challenge, especially in non-endemic areas.

#### 3.7.1. Cardiac Involvement

Involvement of the heart in the form of pericardial effusion, pericarditis, and endocarditis has been reported in many countries like Australia and Malaysia, as well as in travelers to Thailand [114,115,116,117]. Pericardial effusion is usually secondary to pulmonary melioidosis and requires chest X-rays or echocardiographs for detection [117]. Despite pericardiocentesis, few cases can develop recurrent pericardial effusion leading to constrictive pericarditis [114]. Infective endocarditis involving a prosthetic aortic valve has also been reported from Australia in a 69-year-old suffering from melioidosis. Strict adherence to therapy during the intensive and continuation phases obviated the need for valve replacement; however, the patient succumbed subsequently to a stroke-related complication [116].

#### 3.7.2. Mediastinal Lymphadenopathy

Mediastinal lymphadenopathy can also be a presentation in melioidosis, but is more commonly observed in tuberculosis, especially in endemic regions. Radiological imaging may not be conclusive in many cases. Endobronchial ultrasound-guided transbronchial needle aspiration (EBUS-TBNA) is a minimally invasive procedure that has been used for diagnosing melioidosis from infected mediastinal lymph nodes in two cases from India. Both these cases had not revealed any particular pathogen in routine investigations. They were treated successfully and showed no relapses in four months of follow-up [118]. A similar case of mediastinal lymphadenitis with lung mass and left-sided supraclavicular abscess was reported from Mexico. Fine-needle aspiration cytology from the lymph nodes revealed *B. pseudomallei* [119]. A case of ceftazidime resistant *B. pseudomallei* isolated from mediastinal lymph nodes was also reported from Taiwan. Ceftazidime was switched to imipenem after which the patient recovered well [120].

A case of migrating pulmonary infiltration was reported from Korea in a 50-year-old man. He presented with intermittent fever and cough for four weeks and initial chest X-ray and Ct revealed multiple satellite nodules in the left lower and upper lung lobes. The case was thought to tuberculosis, but culture from bronchoscopy washings revealed *B. pseudomallei*. This was, incidentally, the first reported case of Melioidosis from Korea [121].

#### 3.7.3. Lung Empyema

A case of left lung empyema with multiple splenic abscesses was reported from Hong Kong. Repeated drainage and intravenous therapy with imipenem was required for clinical resolution [122]. Cavitary lesions of the lung have also been reported in otherwise healthy individuals with no risk factors. In a case report from Sri Lanka, a young healthy female presented with bilateral cavitary lesions involving all three zones of the lungs. *B. pseudomallei* was isolated from the broncho-alveolar lavage after all other microbiological investigations turned out negative [123]. A similar case was also reported from China where cavitary lesions were accompanied by lung abscesses and osteomyelitis due to melioidosis [124].

#### 3.7.4. Pulmonary Embolism

Pulmonary embolism can also be an uncommon but serious presentation of melioidosis. A case of pulmonary embolism and deep vein thrombosis (DVT) was reported from China and the patient failed to recover despite prompt institution of appropriate antibiotic therapy. The patient was diabetic and involved in farming, thus predisposing him to melioidosis [6]. Pseudoaneurysm of intrathoracic subclavian artery was also reported in a 58-year-old man who presented with sepsis and hoarseness. Blood cultures were positive for *B. pseudomallei* and the aneurysm had to be resected with autogenous vein replacement. The patient recovered after ceftazidime therapy and showed no recurrence upon follow-up after two years [125]. There are also reports of development of pneumatoceles in cases of melioidosis. The case in point was a neonate who presented with septic shock soon after delivery and later succumbed with worsening hypoxia and acidosis [126].

### 3.8. Coinfections

Coinfections of melioidosis with other infectious diseases have been reported in the literature, with the most common being tuberculosis, leptospirosis, leishmaniasis, influenza A, dengue, Japanese encephalitis, and leprosy.

#### 3.8.1. Coinfection with Leptospirosis

Leptospirosis seems, by far, the most commonly reported coinfection, with many similarities to melioidosis in terms of epidemiological and clinical features. Four cases of coinfection of melioidosis and leptospirosis were reported from Pahang, Malaysia in 2010. All four individuals were involved in a search and rescue operation in a recreational forest in Pahang and presented with fever, diarrhea, and myalgia shortly afterwards. *B. pseudomallei* was isolated by blood culture and leptospirosis diagnosis was confirmed by PCR. Despite treatment, there were three fatalities [127]. A similar case of fatal coinfection was reported in a 40-year-old schoolteacher from Malaysia who had a positive history of travel to a freshwater camping site [128]. In India, a case of leptospirosis and melioidosis coinfection was reported in a 42-year-old male who presented with acute respiratory distress syndrome and osteomyelitis. He improved with intravenous meropenem and doxycycline [129]. Another fatal case was reported from Taiwan where a young military personnel was positive for leptospirosis and scrub typhus by serology and *B. pseudomallei* by culture. Despite antibiotic therapy, the patient could not recover and died five days after the onset of fever [130].

#### 3.8.2. Coinfection with Tuberculosis

Tuberculosis and melioidosis coinfections are uncommon, but there have been reports from endemic areas. One report from Brunei Darussalam documented a 64-year-old man with type II diabetes mellitus presenting with a history of chronic cough for the last three weeks. The patient yielded acid-fast bacilli from sputum microscopy and *B. pseudomallei* from sputum culture. He was treated successfully for both over a period of 9 months and showed complete recovery [131]. Another case report showed the coexistence of *B. pseudomallei* and M. tuberculosis in a resected liver abscess from a 49-year-old man from Malaysia. He improved clinically on intravenous medications and anti-tubercular drugs [132]. A report from Thailand showed the coexistence of *Mycobacterium avium* and *B. pseudomallei* from a lymph node aspirate in an HIV-positive Thai female. The patient was managed with intravenous ceftazidime for melioidosis and clarithromycin for *M. avium* and recovered with resolution of fever and abdominal pain [133].

#### 3.8.3. Coinfection with Cutaneous Leishmaniasis

A case of melioidosis and cutaneous leishmaniasis was reported from Sri Lanka. The patient was a 61-year-old diabetic female and presented with fever, hepatosplenomegaly, and an ulcer over the left arm. Blood cultures grew *B. pseudomallei,* and a slit skin smear from the ulcer showed Leishmania amastigotes. She responded to intravenous meropenem and oral co-trimoxazole. The skin ulcer was cauterized and required no further treatment [134]. Coinfection with viral diseases like Japanese encephalitis (JE) and dengue virus have also been reported previously.

#### 3.8.4. Coinfection with Viral Pathogens

In China, a patient with melioidosis was treated with intravenous ceftazidime but failed to respond initially, raising the suspicion of an alternative pathogen. IgM antibodies against JE were detected by ELISA subsequently and the diagnosis of melioidosis was reconfirmed by real-time PCR. The patient received additional supportive therapy and finally responded, with almost complete clinical recovery after three months [135]. A case of dengue–melioidosis coinfection was reported from Brazil in a 28-year-old truck driver who was unsuccessfully treated for sepsis. After death, ascitic fluid culture yielded *B. pseudomallei* and cerebrospinal fluid (CSF) showed dengue virus type I [136]. Secondary bacterial pneumonia caused by *B. pseudomallei* has been reported from Malaysia in a case of influenza A-induced viral pneumonia. The case was managed with intravenous ceftazidime and oral co-trimoxazole [137].

#### 3.8.5. Other Coinfections

Cat-scratch disease is another zoonotic infection which share a number of epidemiological similarities with melioidosis. Coinfection has been reported from Malaysia in a two-year-old boy who had a history of being exposed to cats and contaminated environments. The boy presented with prolonged fever and painless cervical lymphadenopathy, which was initially managed with intravenous ceftazidime and oral azithromycin and then oral co-amoxyclav after being discharged [138]. Association with leprosy has also been reported in the literature. A case of dapsone-induced methemoglobinemia in a 22-year-old leprosy patient was complicated by a concurrent melioidosis infection. The patient’s illness was managed with ceftazidime and co-trimoxazole. The entire treatment duration was laden with complications like co-amoxyclav-induced hypersensitivity, type II lepra reactions; the patient, however, recovered after 12 months of treatment [139]. A case of melioidosis coinfection with Mycobacterium tuberculosis and Salmonella enterica was also demonstrated in a diabetic male presenting with a cervical abscess [140].

### 3.9. Disseminated/Multi-Organ Involvement

A severe and frequently fatal form of melioidosis is called disseminated melioidosis. Individuals with underlying conditions such as diabetes, chronic renal disease, and immunosuppression are more prone to suffer from multi-organ involvement in melioidosis. Hematogenous spread causes disseminated disease, which presents as a multifocal infection involving the central nervous system, pneumonia, visceral abscesses, and osteomyelitis. Its unpredictable presentation frequently delays the start of the right treatment.

In Sri Lanka, a 58-year-old female presented with fever, multiple tender lumps in different parts of the body, erythematous rashes over the skin, headache, arthralgia and loss of appetite lasting 5 weeks. Diagnosis was delayed due to the varied clinical presentation and the patient deteriorated. *B. pseudomallei* was finally confirmed from blood, knee joint, and skin lesion aspirate culture and the patient recovered on intravenous meropenem [141].

Inadequate therapy or misdiagnosis at initial presentation can also give rise to disseminated forms of melioidosis in some cases. In China, a patient with pneumonia where the diagnosis of melioidosis was missed initially, presented after 8 months with left-sided hemiparesis. A brain abscess was detected by CT and pus culture revealed *B. pseudomallei* [142]. Similarly, involvement of multiple bone joints and kidneys was demonstrated in a melioidosis patient by a three-phase bone scan in Kerala, India [143]. Another report from Israel documented disseminated melioidosis in an agricultural worker hailing from Thailand. He presented with multiple subcutaneous abscesses, cervical lymphadenopathy, hepatomegaly, and osteomyelitis [144]. Two cases of disseminated melioidosis associated with hairy cell leukemia were reported in travelers returning from Thailand. They were treated successfully with intravenous ceftazidime and co-trimoxazole with doxycycline [145].

A fatal case of disseminated melioidosis was reported in a 22-year-old male who contracted the disease after a motorbike accident. He developed multiple pustules and succumbed to the infection after 11 days. Incidentally, whole genome sequencing of isolates recovered from different parts of the body revealed fourteen closely related *B. pseudomallei* genotypes, raising the possibility of in vivo diversification of the bacteria during an ongoing infection [146]. Another case with multi-system involvement was reported from Malawi, involving a 16-month-old boy who presented with dactylitis, arthritis, multiple nodules over the face, thorax and limbs with cervical and inguinal lymphadenopathy. The patient recovered on intravenous ceftazidime [147]. Similar cases have been reported wherein diagnosis was often delayed due to multiple conflicting presentations like fever, pneumonia, sepsis, multiple abscesses in different parts of the body, presence of purpuric spots on the skin, association with pregnancy and urinary tract infection, and bone-joint involvement [148,149,150,151].

### 3.10. Other Uncommon Presentations

Apart from these atypical systemic presentations of melioidosis, several other reports have been documented in the literature showing ocular involvement, inflammation of the parotid glands, mimicking malignancy, involvement of head and neck region or in combination with diseases like cystic fibrosis.

#### 3.10.1. Association with Cystic Fibrosis

In the Netherlands, a 38-year-old white male with cystic fibrosis presented with as many as 13 exacerbations of respiratory tract infections between 1992 and 1998 after returning from endemic areas like Indonesia and Thailand. Assumed as, and treated for, *Pseudomonas aeruginosa*, the actual diagnosis could only be made in 1998, when a new screening for *Burkholderia* species was introduced in the laboratory [152]. Another 17-year-old female patient with cystic fibrosis had similar respiratory exacerbations due to *B. pseudomallei* infections. Despite being treated with meropenem, she continued to harbor the bacteria during the two-year follow-up period. Antibody titres also remained high during this period [153]. Similarly, a 14-year-old boy with cystic fibrosis from Malaysia had repeated recurrences of melioidosis over a three-year period and ultimately succumbed to respiratory failure during the fourth year [154]. *B. pseudomallei* was also isolated in conjunction with *Staphylococcus aureus* and *Aspergillus fumigatus* from a 26-year-old female with cystic fibrosis who had history of extensive travel to Southeast Asia prior to diagnosis [155]. This shows that apart from diabetes and chronic renal failure, cystic fibrosis can also be a precursor for melioidosis, especially in people living in or traveling to endemic regions.

#### 3.10.2. Melioidosis in the Head and Neck Region

Some unusual presentations of head and neck melioidosis have also been reported. A case of melioidosis presenting as a nasopharyngeal mass resembling a tumor was reported from Malaysia in a 12-year-old boy. He also suffered from a solitary splenic abscess and cervical lymphadenopathy which yielded *B. pseudomallei* on culture [156]. Another case series from Malaysia reported a similar case of parapharyngeal cellulitis leading to airway obstruction in a 24-year-old pregnant woman from Malaysia. The same series reported three other cases of acute suppurative lymphadenitis, parotod abscess, and chronic suppurative otitis media leading to meningoencephalitis [157]. Suppurative parotitis was also reported in a three-year-old boy who subsequently recovered on ceftazidime and co-trimoxazole [158]. Two cases of suppurative cervical lymphadenitis were reported in returning travelers from Vietnam [159]. A case of disseminated head and neck melioidosis was reported from Australia involving the right parotid, masseter, temporalis and pterygoid muscles, external auditory canal, and involvement of the right middle ear and mastoid cavity [160]. One case of subgaleal abscess was reported from Sri Lanka in a 64-year-old man who responded favorably to ceftazidime and co-trimoxazole [10]. A case of head injury leading to disseminated melioidosis was also reported from Thailand [161].

#### 3.10.3. Lymphadenitis

Inguinal lymphadenitis was also reported in a 53-year-old Sinhalese woman who had been treated for thigh abscesses. She had raised antibody titres against *B. pseudomallei* and was treated with ceftazidime and co-amoxyclav [162]. Cervical lymphadenitis was also reported in a returning traveler from India. Upon investigation, culture yielded *B. pseudomallei* and the patient was treated with intravenous ceftazidime and oral co-trimoxazole [163]. A case of postauricular lymphadenitis was reported in a traveler from Nigeria and blood culture yielded *B. pseudomallei* [164].

#### 3.10.4. Melioidosis Diagnosed as Cancer

A case series from Australia documented seven cases of melioidosis that were wrongly diagnosed as cancer. There were significant delays in diagnosis and three of these patients had already received anti-cancer treatment. All these individuals had comorbidities predisposing them to melioidosis and recovered well with adequate therapy for melioidosis [165].

#### 3.10.5. Ocular Involvement

Ocular presentations in melioidosis are also rare, but have been reported in the literature. A case of sclerokeratitis caused by *B. pseudomallei* was reported from Thailand. The patient had multiple subconjunctival/scleral abscesses and required surgical intervention along with antibiotic therapy for recovery [166]. A case of endophthalmitis caused by *B. pseudomallei* was also reported in a Taiwanese male who was diabetic and had a history of pulmonary tuberculosis. He was treated with systemic and intravitreal ceftazidime [167].

#### 3.10.6. Melioidosis Caused by Imported Spray

A case series from the U.S. reported four cases of melioidosis in residents who had no history of travel to melioidosis-endemic areas. The source of infection was traced back to a particular brand of aromatherapy room spray which was imported from India. The individual isolates were subjected to whole genome sequencing, and it was observed that the isolate from the spray bottle and each of the four cases was identical [168].

## 4. Discussion

The classic presentation of melioidosis includes pneumonia and sepsis. Nonetheless, data from the growing body of clinical literature suggests that atypical manifestations are becoming more widely acknowledged. These include cutaneous, vascular, genitourinary, thoracic, neurological, and musculoskeletal involvement. The diagnosis and start of proper therapy are frequently delayed by these unusual presentations, which commonly resemble other inflammatory or infectious diseases.

Even though it has only been documented in 3–10% of cases, neurological involvement is one of the most debilitating forms of melioidosis. Signs and symptoms like encephalomyelitis, transverse myelitis, brain abscesses, cranial nerve palsies, and even Guillain–Barre like syndromes demonstrate how the pathogen can enter and remain in the central nervous system [20,21]. These cases are frequently misdiagnosed as autoimmune neuropathies, viral encephalitis, or tuberculosis, particularly in non-endemic areas. Results vary greatly based on host comorbidities and the promptness of therapy, but neuroimaging and brain tissue culture seem to be more sensitive for diagnosis than CSF [23].

Additional diagnostic difficulties are brought on by musculoskeletal symptoms, such as pyomyositis, osteomyelitis, septic arthritis, and spinal involvement [43,61]. Especially in endemic areas, these symptoms often resemble tuberculosis, leading to inappropriate empirical treatment [51]. In line with the larger epidemiology of melioidosis, diabetes mellitus is found to be the most prevalent risk factor. Although timely carbapenem or ceftazidime therapy in conjunction with surgery helps many patients recover, cases of septic arthritis or spinal disease have been known to have fatal outcomes.

The symptoms of cutaneous melioidosis can range widely, including necrotizing fasciitis, cellulitis, panniculitis, erythema nodosum, ulcers, and abscesses [70,73,77]. The possibility of latency and reactivation, sometimes decades after initial exposure, makes these forms clinically significant even though they are less frequently fatal. Crucially, it has been demonstrated that oral eradication therapy alone can often effectively treat localized skin disease, setting it apart from the more invasive types.

Although still uncommon, visceral and vascular involvement in melioidosis is dangerous. Hepatic and splenic abscesses are the most prevalent visceral presentations, and they frequently need to be distinguished from pyogenic abscesses due to other causes [87]. Special imaging characteristics, like the “honeycomb” or “necklace” signs, can offer crucial diagnostic hints [95]. Despite intensive surgical and medical treatment, vascular melioidosis, which usually manifests as mycotic aneurysms of the aorta or iliac arteries, has a high fatality rate [100].

The clinical spectrum is further expanded by genitourinary and thoracic manifestations, which include endocarditis, renal disease, pericarditis, mediastinal lymphadenitis, prostatic abscess, and tubo-ovarian abscess [108,110,118]. In atypical melioidosis, the concept of diagnostic ambiguity is reinforced by the fact that many of these presentations resemble more prevalent illnesses. Coinfections with pathogens like Leptospira, Mycobacterium tuberculosis, dengue virus, and Leishmania make diagnosis even more difficult, particularly in tropical areas [127,131]. These incidents warn against depending exclusively on clinical or radiological results and emphasize the need for microbiological confirmation. As the most severe type, disseminated or multi-organ melioidosis involves the skin, bones, viscera, and central nervous system all at the same time due to hematogenous spread [141,146]. Immunosuppression, diabetes, and renal disease are still major contributors to the poor prognosis and spread of the disease.

Apart from these, a large number of other atypical manifestation involving the eyes and the parotid glands, mimicking malignancies, complicating cystic fibrosis, etc., can make melioidosis truly difficult to diagnose and treat [152,156].

### 4.1. Diagnostic Challenges

Because atypical melioidosis can mimic other common infectious or non-infectious diseases, managing it can be very challenging. Neuro-melioidosis can be mistaken for autoimmune neuropathies, viral encephalitis, or tuberculosis; musculoskeletal disorders are frequently mistaken for tubercular osteomyelitis, and cutaneous manifestations can be mistaken for cellulitis, panniculitis, and inflammatory dermatoses [30]. Low clinical suspicion in non-endemic areas exacerbates diagnostic delays. Although microbiological confirmation is still the gold standard, culture yield can be low, especially in patients with central nervous system disease and those already taking antibiotics. Availability of selective media for *B. pseudomallei* and automated culture techniques is a concern for most resource-limited settings. Despite their occasional distinctiveness, imaging features are frequently non-specific. While they have potential, novel diagnostic techniques like molecular detection and lateral flow assays are not yet widely available in all settings.

### 4.2. Strengths and Limitations

The current review combines published literature from both endemic and non-endemic regions to showcase the varied atypical presentations in melioidosis. The current work’s strength is its extensive reach, which compiles information from various systems to give physicians a single, comprehensive resource on atypical disease forms. Additionally, it highlights new trends like latency, reactivation, and how international travel affects disease epidemiology.

Nevertheless, there were some restrictions on the current review. The majority of the literature that is currently available is based on case studies or brief series, which makes it difficult to extrapolate results or calculate incidence rates. The majority of the data originate from Australia and Southeast Asia, which may underrepresent cases in other endemic or developing regions. This is another example of geographical bias. Another issue is publication bias, since cases that are uncommon or severe are more likely to be reported. Comparisons are further complicated by the variations in management strategies and diagnostic standards among studies.

### 4.3. Clinical Implications

In order to prevent misdiagnosis and start the right treatment, clinicians in both endemic and non-endemic areas must have a deeper understanding of the atypical presentations in melioidosis, particularly when they resemble inflammatory disorders, viral infections, or tuberculosis. By regularly considering melioidosis when making differential diagnoses for abscesses, atypical neurological syndromes, and uncommon musculoskeletal infections, better diagnostic accuracy and lower morbidity can be guaranteed. When assessing atypical infections, clinicians should specifically ask about travel and exposure histories in non-endemic areas where awareness is still low. To enhance case detection from a public health standpoint, laboratory personnel must be trained in safe culture handling, and rapid diagnostic assays must be used more widely. The cornerstone of management continues to be early and appropriate antibiotic therapy guided by culture and sensitivity testing; however, when indicated, the integration of surgical interventions is equally important for favorable outcomes.

## 5. Conclusions

The atypical manifestations of melioidosis highlight the remarkable clinical versatility of the bacteria and its capacity to mimic a wide range of infectious and inflammatory conditions. Clinical awareness is necessary to prevent delay in diagnosis and subsequent management, especially in endemic areas and among travelers to endemic areas. Prompt diagnosis and management with carbapenems like imipenem an meropenem are key to reduce morbidity and mortality. Surgical intervention is often required in complicated cases. To better define risk factors, outcomes, and the best management practices for these rare but significant presentations, more multicentric research on sources of infection, risk factors, prognostic indicators, and treatment algorithms are required.

## Figures and Tables

**Table 1:** System-wise articles used for the review, showing the major presentations in each system.

Sl. No.	System Affected	Common Presentations	Total Number of Studies Included
1	Neurological	Brain abscess, encephalomyelitis, GBS-like syndrome.	24
2	Musculoskeletal	Osteomyelitis, myositis, septic arthritis.	28
3	Genitourinary	Tubo-ovarian abscess, prostatic abscess.	8
4	Cutaneous	Erythema nodosum, necrotizing fasciitis, skin ulcers.	18
5	Thoracic/Mediastinal	Mediastinal lymphadenopathy, chronic pneumonia, PE/DVT.	14
6	Visceral	Abscess, organomegaly.	14
7	Vascular	Aneurysms.	10
8	Coinfections	Leptospirosis, tuberculosis, leishmaniasis.	14
9	Disseminated	Multi-organ involvement.	12
10	Others	Head and neck, melioidosis in cystic fibrosis patients, lymphatic, malignancy-like, aromatherapy, ocular.	18

## Data Availability

All articles included in the current manuscript are publicly available in PubMed.

## Abbreviations

The following abbreviations are used in this manuscript:

DVT	Deep vein thrombosis
PCR	Polymerase chain reaction
JE	Japanese encephalitis
CSF	Cerebrospinal fluid
CT	Computed tomography

## References

[1] Jabbar Z., Currie B.J. (2013). Melioidosis and the kidney. Nephrology.

[2] Gupta N., Malla S., Boodman C., Kumar T.P., Varma M., Mukhopadhyay C. (2025). Abscesses due to Melioidosis: A case-based review. Curr. Res. Microb. Sci..

[3] Hadano Y. (2018). Imported melioidosis in Japan: A review of cases. Infect. Drug Resist..

[4] Raja N.S., Scarsbrook C. (2016). *Burkholderia Pseudomallei* Causing Bone and Joint Infections: A Clinical Update. Infect. Dis. Ther..

[5] Tantawarak N., Techasatian L., Ungarreevittaya P., Kosalaraksa P., Lumbiganon P. (2024). Case Report: Melioidosis-Related Severe Cutaneous Features of Erythema Nodosum in a Thai Child. Am. J. Trop. Med. Hyg..

[6] Wu H., Huang D., Wu B., Pan M., Lu B. (2019). Fatal deep venous thrombosis and pulmonary embolism secondary to melioidosis in China: Case report and literature review. BMC Infect. Dis..

[7] Shrestha N., Adhikari M., Pant V., Baral S., Shrestha A., Basnyat B., Sharma S., Sherchand J.B. (2019). Melioidosis: Misdiagnosed in Nepal. BMC Infect. Dis..

[8] Nernsai P., Sophonsritsuk A., Lertvikool S., Jinawath A., Chitasombat M.N. (2018). A case report of Tubo-ovarian abscess caused by *Burkholderia pseudomallei*. BMC Infect. Dis..

[9] Kannan S., Singh S., Earny V.A., Chowdhury S., Ashiq M., Eshwara V.K., Mukhopadhyay C., Kaur H. (2025). Two Decades of Melioidosis in India: A Comprehensive Epidemiological Review. Pathogens.

[10] Dalugama C., Tennegedara A., Gawarammana I.B. (2018). De novo subgaleal abscess—A rare presentation of melioidosis: A case report. J. Med. Case Rep..

[11] Chowdhury S., Barai L., Afroze S.R., Ghosh P.K., Afroz F., Rahman H., Ghosh S., Hossain M.B., Rahman M.Z., Das P. (2022). The Epidemiology of Melioidosis and Its Association with Diabetes Mellitus: A Systematic Review and Meta-Analysis. Pathogens.

[12] Owen W., Smith S., Kuruvath S., Anderson D., Hanson J. (2021). Melioidosis of the central nervous system; A potentially lethal impersonator. IDCases.

[13] Harris J., Smith S., Ng S.Z., Sinha A., Hanson J. (2022). Case Report: Disseminated *Burkholderia pseudomallei* with Acute Suppurative Thyroiditis and Abscess Formation. Am. J. Trop. Med. Hyg..

[14] Wongyikul P., Klangbud W.K., Chatatikun M., Phinyo P. (2025). Co-Infections and Their Prognostic Impact on Melioidosis Mortality: A Systematic Review and Individual Patient Data Meta-Analysis. Epidemiologia.

[15] Songsri J., Chatatikun M., Wisessombat S., Mala W., Phothaworn P., Senghoi W., Palachum W., Chanmol W., Intakhan N., Chuaijit S. (2024). Diagnostic accuracy of automation and non-automation techniques for identifying *Burkholderia pseudomallei*: A systematic review and meta-analysis. J. Infect. Public Health.

[16] Birnie E., Virk H.S., Savelkoel J., Spijker R., Bertherat E., Dance D.A.B., Limmathurotsakul D., Devleesschauwer B., Haagsma J.A., Wiersinga W.J. (2019). Global burden of melioidosis in 2015: A systematic review and data synthesis. Lancet Infect. Dis..

[17] Raina S., Gautam D., Kumar R., Sisodia K., Mukhopadhyay C., Kaur H., Ashiq M., Saksena R. (2025). Unearthing the burden of melioidosis in North India—An emerging threat in a non-endemic region. Curr. Res. Microb. Sci..

[18] Bommakanti K., Ankathi P., Uma P., Malladi S., Laxmi V. (2010). Cerebral abscess and calvarial osteomyelitis due to *Burkholderia pseudomallei*. Neurol. India.

[19] Ekka A.S., Mohideen M., Kesavan S. (2017). Neuromelioidosis Masquerading as Acute Demyelinating Encephalomyelitis. Indian Pediatr..

[20] White M.E., Hunt J., Connell C., Langdon K. (2016). Paediatric neurological melioidosis: A rehabilitation case report. Rural Remote Health.

[21] Nandasiri S., Wimalaratna H., Manjula M., Corea E. (2012). Transverse myelitis secondary to melioidosis: A case report. BMC Infect. Dis..

[22] Varkey Maramattom B., Rathish B. (2021). Case Report: Ascending Myelo-Encephalitis after a Penetrating Injury to the Foot: An Atypical Case of Neuromelioidosis. Am. J. Trop. Med. Hyg..

[23] Vithoosan S., Kumarasiri A., Vithanage N.M., Senanayake B. (2022). Case report long segment myelitis secondary to neuro melioidosis. BMC Neurol..

[24] Antony T., Moorthy S., Narayanaswamy A., Arthur P. (2017). Melioidosis presenting as septicaemia and facial nerve palsy. BMJ Case Rep..

[25] Matthews R.J., Smith S., Wilson I., Tjahjono R., Young S., Hanson J. (2023). Case Report: Vagal Nerve Neuritis Associated with Pulmonary Melioidosis Provides Potential Insights into the Pathophysiology of Neuromelioidosis. Am. J. Trop. Med. Hyg..

[26] Chatterjee A., Saravu K., Mukhopadhyay C., Chandran V. (2021). Neurological Melioidosis Presenting as Rhombencephalitis, Optic Neuritis, and Scalp Abscess with Meningitis: A Case Series from Southern India. Neurol. India.

[27] Wijekoon P.W., Bandara K.A., Kailainathan A., Chandrasiri N.S., Hapuarachchi C.T. (2016). Guillaine-barre syndrome; a rare complication of melioidosis. a case report. BMC Infect. Dis..

[28] Koh E.J., Tan K.N., Chan Z.W., Candice Wong H.Y., Chin M.L., Lee T.C. (2023). A New Association of Guillain Barre Syndrome in a Patient with Central Nervous System Melioidosis. J. Ayub Med. Coll. Abbottabad.

[29] Chlebicki M.P., Kurup A., Sin Y.K. (2008). *Burkholderia pseudomallei* meningitis following inadequate treatment of melioidotic mycotic aneurysm. Singap. Med. J..

[30] Mishra B., Vishnu V.Y., Bhatia R., Garg A., Doddamani R.S., Singh P., Chand Sharma M., Singh M.B., Rajan R., Gupta A. (2021). Case Report: Isolated Central Nervous System Melioidosis from a Non-Endemic Area. Am. J. Trop. Med. Hyg..

[31] Nayak R., Patel B., Raju K. (2018). Chronic pachymenigitis with dural venous sinus thrombosis: An unusual presentation of cranial melioidosis. Neurol. India.

[32] Arif M.A., Abid M.H., Renganathan R., Siddiqui K.A. (2015). Central and peripheral nervous system involvement in neuromelioidosis. BMJ Case Rep..

[33] Shetty N.S., Karikal A., Radhakrishnan S.M. (2022). Melioidosis: An unusual diagnosis for deep temporal and masticatory space infection following trauma. BMJ Case Rep..

[34] Saravu K., Kadavigere R., Shastry A.B., Pai R., Mukhopadhyay C. (2015). Neurologic melioidosis presented as encephalomyelitis and subdural collection in two male labourers in India. J. Infect. Dev. Ctries..

[35] Chen G.B., Tuan S.H., Chen L.H., Lin W.S. (2018). Neurological melioidosis (*Burkholderia pseudomallei*) in a chronic psychotic patient treated with antipsychotics: A case report. Medicine.

[36] Zhan Y., Wu Y., Li Q., Yu A. (2017). Neuromelioidosis: A series of seven cases in Hainan province, China. J. Int. Med. Res..

[37] Vestal M.L., Wong E.B., Milner D.A., Gormley W.B., Dunn I.F. (2013). Cerebral melioidosis for the first time in the western hemisphere. J. Neurosurg..

[38] Shobhana A., Datta A., Trivedi S. (2022). CNS Melioidosis: A Diagnostic Challenge. Neurol. India.

[39] Vithana S.M.P., Chathuranga L.S., Jayasinghe S., Udayakumara E.A.D. (2022). A rare case of melioidosis presenting as myositis in Sri Lanka. BMC Infect. Dis..

[40] Vaid T., Rao K., Hande H.M. (2015). An intriguing case of locked jaw secondary to melioidosis. BMJ Case Rep..

[41] Jayarajah U., Arulanantham A., Koculen V., Palkumbura C., Faleel A., Sooriyarachchi R. (2020). *Burkholderia pseudomallei* peri-prosthetic infection following medial malleolar internal fixation: A case report. BMC Infect. Dis..

[42] Boyer P.N., Woods M.L. (2020). *Burkholderia pseudomallei* sepsis with osteoarticular melioidosis of the hip in a patient with diabetes mellitus. BMJ Case Rep..

[43] Frincy K.B., Biswajyoti B., Saikia S., Baruah M.P., Devi U. (2020). *Burkholderia pseudomallei* septic arthritis in Type-2 diabetes mellitus patients: Report of two cases. Indian J. Med. Microbiol..

[44] Melikyan G., Badawi M., Akhtar N., Elsetouhy A.H. (2013). Case of paraspinal collection due to *Burkholderia pseudomallei*. BMJ Case Rep..

[45] Weerasinghe N.P., Herath H.M.M., Liyanage T.M.U. (2018). Isolated septic arthritis of hip joint: A rare presentation of melioidosis. A case report. BMC Res. Notes.

[46] Mukhopadhyay C., Dey A., Bairy I. (2007). Atypical presentations of melioidosis as emerging threat: A case report. Indian J. Pathol. Microbiol..

[47] Chang C.Y. (2021). Pyomyositis masquerading as suprapubic mass: An unusual presentation of melioidosis. Ceylon Med. J..

[48] Miksić N.G., Alikadić N., Lejko T.Z., Andlovic A., Knific J., Tomazic J. (2007). Osteomyelitis of parietal bone in melioidosis. Emerg. Infect. Dis..

[49] Stewart T., Engelthaler D.M., Blaney D.D., Tuanyok A., Wangsness E., Smith T.L., Pearson T., Komatsu K.K., Keim P., Currie B.J. (2011). Epidemiology and investigation of melioidosis, Southern Arizona. Emerg. Infect. Dis..

[50] Jayawardena N., Ralapanawa U., Kumarihamy P., Jayalath T., Abeygunawardana S.P., Dissanayake N., Dissanayake P., Udupihille J., Ratnatunga N., Dalugama C. (2019). Infective myositis, an uncommon presentation of melioidosis: A case report and review of the literature. J. Med. Case Rep..

[51] Kundangar R.S., Bhat S.N., Mohanty S.P. (2017). Melioidosis mimicking tubercular cold abscess. BMJ Case Rep..

[52] Pande A., Nambi P.S., Pandian S., Subramanian S., Ghosh S. (2018). Melioidosis mimicking tuberculous vertebral osteitis: Case report and review of literature. Neurol. India.

[53] Garg R., Shaw T., Bhat S.N., Mukhopadhyay C. (2018). Melioidosis: The great mimicker presenting as spondylodiscitis. BMJ Case Rep..

[54] Prasad G.L., Nair R.P., Menon G.R. (2017). Intracranial melioidosis: First report in a human immunodeficiency virus positive individual manifesting as cranial osteomyelitis. Neurol. India.

[55] Kuan Y.C., How S.H., Ng T.H., Fauzi A.R. (2010). The man with the boggy head: Cranial melioidosis. Singap. Med. J..

[56] Xiao L., Zhou T., Chen J., Hu Y., Zheng Y. (2022). Paravertebral abscess and bloodstream infection caused by *Burkholderia pseudomallei* after acupuncture: A case report. BMC Complement. Med. Ther..

[57] Mitchell P.K., Campbell C., Montgomery M.P., Paoline J., Wilbur C., Posivak-Khouly L., Garafalo K., Elrod M., Liu L., Weltman A. (2017). Notes from the Field: Travel-Associated Melioidosis and Resulting Laboratory Exposures—United States, 2016. MMWR Morb. Mortal. Wkly. Rep..

[58] Subran B., Ackermann F., Watin-Augouard L., Rammaert B., Rivoisy C., Vilain D., Canzi A.M., Kahn J.E. (2013). Melioidosis in a European traveler without comorbidities: A case report and literature review. Int. J. Infect. Dis..

[59] Mukhopadhyay C., Dey A., Sugandhi Rao P., Pandey V., Sripathi Rao P. (2007). Aetiology and management of chronic granulomatous osteomyelitis: Look before you leap. Singap. Med. J..

[60] Mabayoje D.A., Kenna D.T.D., Dance D.A.B., NicFhogartaigh C. (2022). Melioidosis Manifesting as Chronic Femoral Osteomyelitis in Patient from Ghana. Emerg. Infect. Dis..

[61] Yazdanpanah Y., Lemaire X., Senneville E., Delcey V., Viget N., Mouton Y., Ajana F., Dubreuil L. (2002). Melioidotic osteomyelitis of the femur occurring in a traveler. J. Travel Med..

[62] Ng W.M., Kwan M.K., Merican A.M. (2006). Melioidotic osteomyelitis treated with antibiotic-calcium hydroxyapatite composite: Case report with four-year follow-up. Singap. Med. J..

[63] Conrad A., Valour F., Ferry T., Ader F. (2016). Multifocal melioidosis with femoral osteomyelitis in a healthy 25-year-old traveller. BMJ Case Rep..

[64] Kow A.W., Lee K.B., Wong Y.S. (2005). Musculoskeletal melioidosis masquerading as diabetic amyotrophy. Singap. Med. J..

[65] Shetty H.S., Mallela A.R., Shastry B.A., Acharya V. (2015). Parietal bone osteomyelitis in melioidosis. BMJ Case Rep..

[66] Soo K.C., Lee K.S., Ooi S.Y., Darwina A., Sannasey S., Lee H.G. (2021). Disseminated Melioidosis with Spinal Intraosseous Abscess. Med. J. Malays..

[67] Pérez M., Currea M., Martínez C., Pérez J., Arango Á. (2021). Prosthetic valve endocarditis in melioidosis. Case report. Rev. Chil. Infectol..

[68] Zainal Abidin I., Syed Tamin S., Huat Tan L., Chong W.P., Azman W. (2007). Pacemaker infection secondary to *Burkholderia pseudomallei*. Pacing Clin. Electrophysiol..

[69] Wootton C.I., Elliott I.A., Sengdetkha D., Vongsouvath M., Phongmany S., Dance D.A. (2013). Melioidosis: An unusual cause of recurrent buttock abscesses. Clin. Exp. Dermatol..

[70] Stavrou C., Veraitch O., Morris-Jones S., Walker S.L. (2021). Leg ulceration due to cutaneous melioidosis in a returning traveller. BMJ Case Rep..

[71] Ezzedine K., Malvy D., Steels E., De Dobbeeler G., Struelens M., Jacobs F., Heenen M. (2007). Imported melioidosis with an isolated cutaneous presentation in a 90-year-old traveller from Bangladesh. Bull. Soc. Pathol. Exot..

[72] Dan M., Taran D. (2015). Melioidosis of the Skin in an Israeli Traveler Returning from Thailand. Isr. Med. Assoc. J..

[73] Hughes S., Loughenbury F., Richards A., Easom N. (2021). Cutaneous thigh abscess secondary to melioidosis: A rare cause for a common presentation. BMJ Case Rep..

[74] Palit A., Rahman M., Rahman M.M., Akhtar Z., Rahman M.Z., Alam M., Deb A.S., Chowdhury S., Rahman M.M., Das P. (2025). Melioidosis with venous thrombosis and cellulitis in the left lower limb: A case report. J. Med. Case Rep..

[75] Yongpisarn T., Santanirand P., Chairanaicharoen S., Rattananukrom T. (2025). A rare case of cutaneous melioidosis manifesting as infective panniculitis: A case report. BMC Infect. Dis..

[76] Vithoosan S., Thanushah B., Piranavan P., Gamlaksha D., Karunatilake H., Jayanaga A. (2019). A rare case of Sweet syndrome secondary to melioidosis. BMC Dermatol..

[77] Diolombi M., Seneviratne M., Norton R. (2020). Case Report: Erythema Nodosum and Melioidosis: An Unreported Association. Am. J. Trop. Med. Hyg..

[78] Tan P.Y., Goh J.Y. (2022). Erythema Nodosum—An atypical presentation of melioidosis. Rev. Soc. Bras. Med. Trop..

[79] Wang Y.S., Wong C.H., Kurup A. (2003). Cutaneous melioidosis and necrotizing fasciitis caused by *Burkholderia pseudomallei*. Emerg. Infect. Dis..

[80] Hill A.A., Mayo M., Kaestli M., Price E.P., Richardson L.J., Godoy D., Spratt B.G., Currie B.J. (2013). Melioidosis as a consequence of sporting activity. Am. J. Trop. Med. Hyg..

[81] Ngauy V., Lemeshev Y., Sadkowski L., Crawford G. (2005). Cutaneous melioidosis in a man who was taken as a prisoner of war by the Japanese during World War II. J. Clin. Microbiol..

[82] Meckenstock R., Therby A., Marque-Juillet S., Monnier S., Khau D., Pangon B., Greder-Belan A. (2012). Cutaneous melioidosis in adolescent returning from Guadeloupe. Emerg. Infect. Dis..

[83] Fung W.K., Tam S.C., Ho K.M., Lam P., Lo K.K. (2001). Porphyria cutanea tarda and melioidosis. Hong Kong Med. J..

[84] Smith S., Hanson J. (2019). Successful Treatment of Localized Cutaneous Melioidosis with Oral Antibiotic Therapy Alone. Am. J. Trop. Med. Hyg..

[85] Bodilsen J., Langgaard H., Nielsen H.L. (2015). Cutaneous melioidosis in a healthy Danish man after travelling to South-East Asia. BMJ Case Rep..

[86] Kuijpers S.C., Klouwens M., de Jong K.H., Langeslag J.C.P., Kuipers S., Reubsaet F.A.G., van Leeuwen E.M.M., de Bree G.J., Hovius J.W., Grobusch M.P. (2021). Primary cutaneous melioidosis acquired in Nepal—Case report and literature review. Travel Med. Infect. Dis..

[87] Chang C.Y. (2023). *Burkholderia pseudomallei* As The Predominant Cause of Splenic Abscess in Kapit, Sarawak, Malaysian Borneo. J. Ayub Med. Coll. Abbottabad.

[88] Chang C.Y., Lee H.L. (2023). A Fatal Case of Persistent *Burkholderia pseudomallei* Bacteraemia with Severe Pneumonia And Splenic Abscess. J. Ayub Med. Coll. Abbottabad.

[89] Chen H., Hu Z.Q., Fang Y., Lu X.X., Li L.D., Li Y.L., Mao X.H., Li Q. (2018). A case report: Splenic abscess caused by *Burkholderia pseudomallei*. Medicine.

[90] Lin C.Y., Chen T.C., Lu P.L., Lin W.R., Chen Y.H. (2007). Melioidosis presenting with isolated splenic abscesses: A case report. Kaohsiung J. Med. Sci..

[91] Gupta A.P., Halder R., Chakraborty M., Chakraborty P.P. (2021). Isolated splenic abscess due to melioidosis in type 1 diabetes mellitus: Laboratory diagnosis of *Burkholderia pseudomallei* in resource-restricted setting. BMJ Case Rep..

[92] Liu Y., Zong Z. (2020). Prolonged intermittent fever and massive splenomegaly in a miner working in the tropical jungle, China. PLoS Negl. Trop. Dis..

[93] Chow T.K., Eu L.C., Chin K.F., Ong K.C., Pailoor J., Vadivelu J., Wong K.T. (2016). Incidental Splenic Granuloma Due to *Burkholderia pseudomallei*: A Case of Asymptomatic Latent Melioidosis?. Am. J. Trop. Med. Hyg..

[94] Yuan Y., Yao Z., Xiao E., Zhang J., Wang B., Ma B., Li Y., Yan W., Wang S., Ma Q. (2019). The first imported case of melioidosis in a patient in central China. Emerg. Microbes Infect..

[95] Ong S.C.L., Alemam M.M.M., Zakaria N.A., Abdul Halim N.A. (2017). Honeycomb and necklace signs in liver abscesses secondary to melioidosis. BMJ Case Rep..

[96] Lee Y.L., Lee S.S., Tsai H.C., Chen Y.S., Wann S.R., Kao C.H., Liu Y.C. (2006). Pyogenic liver abscess caused by *Burkholderia pseudomallei* in Taiwan. J. Formos. Med. Assoc..

[97] Chong V.H., Lim K.S., Sharif F. (2010). Pancreatic involvement in melioidosis. Jop.

[98] Wong K.W. (2013). Melioidosis and peritoneal dialysis related peritonitis. Med. J. Malays..

[99] Kiertiburanakul S., Sungkanuparph S., Kositchiwat S., Vorachit M. (2002). *Burkholderia pseudomallei*: Abscess in an unusual site. J. Postgrad. Med..

[100] Wu H., Wang X., Zhou X., Wu Z., Wang Y., Pan M., Lu B. (2020). Mycotic aneurysm secondary to melioidosis in China: A series of eight cases and a review of literature. PLoS Negl. Trop. Dis..

[101] Azizi Z.A., Yahya M., Lee S.K. (2005). Melioidosis and the vascular surgeon: Hospital Kuala Lumpur experience. Asian J. Surg..

[102] Boyle R., Withey G., Smith S., Hanson J. (2024). Mycotic aneurysms due to *Burkholderia pseudomallei* in Far North Queensland, tropical Australia: A case series and review of the literature. Acta Trop..

[103] Luo C.Y., Ko W.C., Lee H.C., Yang Y.J. (2003). Relapsing melioidosis as cause of iliac mycotic aneurysm: An indigenous case in Taiwan. J. Vasc. Surg..

[104] Rao J., Kaushal A.S., Hoong C.K. (2009). Abdominal aortic pseudoaneurysm secondary to melioidosis. Asian J. Surg..

[105] Bodilsen J., Vammen S., Fuursted K., Hjort U. (2014). Mycotic aneurysm caused by *Burkholderia pseudomallei* in a previously healthy returning traveller. BMJ Case Rep..

[106] Amezyane T., Lecoules S., Algayres J.P. (2010). Mycotic iliac aneurysm associated with *Burkholderia pseudomallei*. Int. J. Infect. Dis..

[107] Porter M.C., Pennell C.E., Woods P., Dyer J., Merritt A.J., Currie B.J. (2018). Case Report: Chorioamnionitis and Premature Delivery due to *Burkholderia pseudomallei* Infection in Pregnancy. Am. J. Trop. Med. Hyg..

[108] Hesstvedt L., Wilhelmsen M., Mengshoel A.T., Dyrhol-Riise A.M. (2011). Two Norwegian patients with melioidosis presenting with bacteraemia and splenic and prostatic abscesses. J. Travel Med..

[109] Naganathan K., Pillai S.B., Kumar P., Hegde P. (2014). Whitmore’s disease: An uncommon urological presentation. BMJ Case Rep..

[110] Demar M., Ferroni A., Dupont B., Eliaszewicz M., Bourée P. (2005). Suppurative epididymo-orchitis and chronic prostatitis caused by *Burkholderia pseudomallei*: A case report and review. J. Travel Med..

[111] Ng T.H., How S.H., Amran A.R., Razali M.R., Kuan Y.C. (2009). Melioidotic prostatic abscess in Pahang. Singap. Med. J..

[112] Dingle T.C., Butler-Wu S.M., Abbott A.N. (2014). Accidental exposure to *Burkholderia pseudomallei* in the laboratory in the era of matrix-assisted laser desorption ionization-time of flight mass spectrometry. J. Clin. Microbiol..

[113] Bousquet A., Gueudet T., Biot F., Bigaillon C., Gominet M., Mérens A. (2020). Accidental exposure to *Burkholderia pseudomallei*: Awareness is also needed for urine specimens. Clin. Microbiol. Infect..

[114] Lu H.T., Ramsamy G., Lee C.Y., Syed Hamid S.R.G., Kan F.K., Nordin R.B. (2018). A Case of Constrictive Pericarditis Associated with Melioidosis in an Immunocompetent Patient Treated by Pericardiectomy. Am. J. Case Rep..

[115] Schultze D., Müller B., Bruderer T., Dollenmaier G., Riehm J.M., Boggian K. (2012). A traveller presenting with severe melioidosis complicated by a pericardial effusion: A case report. BMC Infect. Dis..

[116] Pownell C., Marsden B.E., Lam W., Smith S., Hanson J. (2025). Prosthetic valve infective endocarditis due to *Burkholderia pseudomallei*: A case report and review of the literature. Acta Trop..

[117] Yew K.L., Ng T.H., How S.H., Kuan Y.C. (2011). The bug and the big heart --melioidotic pericardial effusion. Med. J. Malays..

[118] Rahman K.K.M., Bhuniya S., Behera B., Mohapatra P.R., Kumar R., Radhakrishnan A. (2020). Utility of endobronchial ultrasound guided trans-bronchial needle aspiration in diagnosis of melioidosis—Case series and review of literature. Monaldi Arch. Chest Dis..

[119] Truong K.K., Moghaddam S., Al Saghbini S., Saatian B. (2015). Case of a lung mass due to melioidosis in Mexico. Am. J. Case Rep..

[120] Kung C.T., Lee C.H., Li C.J., Lu H.I., Ko S.F., Liu J.W. (2010). Development of ceftazidime resistance in *Burkhoderia pseudomallei* in a patient experiencing melioidosis with mediastinal lymphadenitis. Ann. Acad. Med. Singap..

[121] Lee S.W., Yi J., Joo S.I., Kang Y.A., Yoon Y.S., Yim J.J., Yoo C.G., Han S.K., Shim Y.S., Kim E.C. (2005). A case of melioidosis presenting as migrating pulmonary infiltration: The first case in Korea. J. Korean Med. Sci..

[122] Tsang T.Y., Lai S.T. (2001). A case of thoracic empyema due to suppurative melioidosis. Hong Kong Med. J..

[123] Fonseka C.L., Galappaththi S.R., Illagatilaka A., Dasanayake D., Tissera N. (2016). Acute pulmonary melioidosis presenting with multiple bilateral cavitary lesions in a healthy young adult: An authentic case report from Sri Lanka. BMC Res. Notes.

[124] Huang L., Yang Z., Zhou X.P., Wu J.R. (2018). *Burkholderia pseudomallei* infection presenting with a lung abscess and osteomyelitis in an adult man: A case report. Medicine.

[125] Schindler N., Calligaro K.D., Dougherty M.J., Diehl J., Modi K.H., Braffman M.N. (2002). Melioidosis presenting as an infected intrathoracic subclavian artery pseudoaneurysm treated with femoral vein interposition graft. J. Vasc. Surg..

[126] Nivedhana S., Rajendran S. (2016). Neonatal Melioidosis with Pneumatoceles. Indian Pediatr.

[127] Hin H.S., Ramalingam R., Chunn K.Y., Ahmad N., Ab Rahman J., Mohamed M.S. (2012). Fatal co-infection—Melioidosis and leptospirosis. Am. J. Trop. Med. Hyg..

[128] Mohd Ali M.R., Mohamad Safiee A.W., Thangarajah P., Fauzi M.H., Muhd Besari A., Ismail N., Yean Yean C. (2017). Molecular detection of leptospirosis and melioidosis co-infection: A case report. J. Infect. Public Health.

[129] Panigrahi M.K., Bal S.K., Tripathy T.P., Moorthy A., Mohanty S.K., Mahapatra A., Bhuniya S. (2024). Leptospirosis and melioidosis coinfection presenting as acute respiratory distress syndrome and osteomyelitis: Case report and systematic review. J. Infect. Dev. Ctries..

[130] Lu P.L., Tseng S.H. (2005). Fatal septicemic melioidosis in a young military person possibly co-infected with Leptospira interrogans and Orientia tsutsugamushi. Kaohsiung J. Med. Sci..

[131] Rubel A.R., Mani B.I., Kishore P.V., Chong V.H. (2022). Pulmonary tuberculosis and melioidosis coinfection in Brunei Darussalam: The importance of awareness and screening. West. Pac. Surveill. Response J..

[132] Azali H.Y., Norly S., Wong L.M., Tan K.S., Safian N.M. (2007). Liver abscess caused by tuberculosis and melioidosis. Asian J. Surg..

[133] Pumpradit W., Ariyoshi K., Petkanchanapong W., Wichukchinda N., Chaiprasert A., Rojanawat A., Sawanpanyalert P., Pathipvanich P. (2006). *Mycobacterium avium* and *Burkholderia pseudomallei* (Melioidosis) coinfection in an HIV-positive patient. Asian Pac. J. Allergy Immunol..

[134] Kahandawaarachchi I.C.I., Premawansa G.S., Warnasuriya W., Dassanayake M., Corea E. (2017). A case report of co-infection of Melioidosis and cutaneous Leishmaniasis. BMC Infect. Dis..

[135] Li X.Y., Ke B.X., Chen C.N., Xiao H.L., Liu M.Z., Xiong Y.C., Bai R., Chen J.D., Ke C.W. (2018). First co-infection case of melioidosis and Japanese encephalitis in China. BMC Infect. Dis..

[136] Macedo R.N., Rocha F.A., Rolim D.B., Vilar D.C., Araújo F.M., Vieira N.N., Teixeira J.R., Carvalho M.C., Oliveira F.G., Cavalcanti L.P. (2012). Severe coinfection of melioidosis and dengue fever in Northeastern Brazil: First case report. Rev. Soc. Bras. Med. Trop..

[137] Tan W.F., Lee H.G. (2021). Concurrent Influenza A and Pulmonary Melioidosis in pregnancy. Med. J. Malays..

[138] Azri M., Ismail I.H. (2022). First documented co-infection case of cat-scratch disease and melioidosis in Malaysia: A cause of undifferentiated prolonged febrile illness. Med J Malays..

[139] Tang A.S.O., Wong Q.Y., Yeo S.T., Ting I.P.L., Lee J.T.H., Fam T.L., Chew L.P., Chua H.H., Muniandy P. (2021). Challenges in Managing a Lepromatous Leprosy Patient Complicated with Melioidosis Infection, Dapsone-Induced Methemoglobinemia, Hemolytic Anemia, and Lepra Reaction. Am. J. Case Rep..

[140] Sulaiman H., Ponnampalavanar S., Mun K.S., Italiano C.M. (2013). Cervical abscesses due to co-infection with *Burkholderia pseudomallei*, Salmonella enterica serovar Stanley and Mycobacterium tuberculosis in a patient with diabetes mellitus. BMC Infect. Dis..

[141] Pathirage M.M.K., Kularatne S.A.M., Weerakoon K.G. (2020). Melioidosis after a long silence in Sri Lanka: An environmental hazard and dilemma in diagnosis, with recovery and longitudinal follow-up for 13 years: A case report. J. Med. Case Rep..

[142] Huang W.Y., Wu G., Chen F., Li M.M., Li J.J. (2018). Multi-systemic melioidosis: A clinical, neurological, and radiological case study from Hainan Province, China. BMC Infect. Dis..

[143] Subramanyam P., Palaniswamy S.S. (2014). Multifocal bone and visceral melioidosis in a cirrhotic patient identified by (99m)Tc MDP bone scan. Am. J. Trop. Med. Hyg..

[144] Cahn A., Koslowsky B., Nir-Paz R., Temper V., Hiller N., Karlinsky A., Gur I., Hidalgo-Grass C., Heyman S.N., Moses A.E. (2009). Imported melioidosis, Israel, 2008. Emerg. Infect. Dis..

[145] Rossi B., Epelboin L., Jauréguiberry S., Lecso M., Roos-Weil D., Gabarre J., Grenier P.A., Bricaire F., Caumes E. (2013). Melioidosis and hairy cell leukemia in 2 travelers returning from Thailand. Emerg. Infect. Dis..

[146] Limmathurotsakul D., Holden M.T., Coupland P., Price E.P., Chantratita N., Wuthiekanun V., Amornchai P., Parkhill J., Peacock S.J. (2014). Microevolution of *Burkholderia pseudomallei* during an acute infection. J. Clin. Microbiol..

[147] Katangwe T., Purcell J., Bar-Zeev N., Denis B., Montgomery J., Alaerts M., Heyderman R.S., Dance D.A., Kennedy N., Feasey N. (2013). Human melioidosis, Malawi, 2011. Emerg. Infect. Dis..

[148] Annamalai A.K., Padmini K. (2019). Melioidosis. Indian J. Med. Res..

[149] Mohapatra A., Agarwala P., Sirigiri H.P., Das P. (2024). Disseminated melioidosis-challenge to routine antibiotic therapy: A case report. J. Med. Case Rep..

[150] Chang C.Y., Lau N.L.J., Currie B.J., Podin Y. (2020). Disseminated melioidosis in early pregnancy—An unproven cause of foetal loss. BMC Infect. Dis..

[151] Wijewickrama P.S.A., Weerakoon R. (2017). Acute disseminated melioidosis giving rise to pneumonia and renal abscesses complicated with thrombotic thrombocytopenic purpura in a post partum woman: A case report. BMC Res. Notes.

[152] Schülin T., Steinmetz I. (2001). Chronic melioidosis in a patient with cystic fibrosis. J. Clin. Microbiol..

[153] Barth A.L., de Abreu E.S.F.A., Hoffmann A., Vieira M.I., Zavascki A.P., Ferreira A.G., da Cunha L.G., Albano R.M., de Andrade Marques E. (2007). Cystic fibrosis patient with *Burkholderia pseudomallei* infection acquired in Brazil. J. Clin. Microbiol..

[154] Mariappan V., Thavagnanam S., Vellasamy K.M., Teh C.J.S., Atiya N., Ponnampalavanar S., Vadivelu J. (2018). Relapse of chronic melioidosis in a paediatric cystic fibrosis patient: First case report from Malaysia. BMC Infect. Dis..

[155] Hettiarachchi I.T., Duckers J., Lau D., Dhillon R. (2019). The Brief Case: A Traveler’s Tale-*Burkholderia pseudomallei* Infection in a Cystic Fibrosis Patient. J. Clin. Microbiol..

[156] Mohan A., Yeong L.C., Kumarasamy G., Manan K. (2023). Nasopharyngeal melioidosis: A case report. J. Infect. Dev. Ctries..

[157] Lim W.K., Gurdeep G.S., Norain K. (2001). Melioidosis of the head and neck. Med. J. Malays..

[158] Shivbalan S., Reddy N., Tiru V., Thomas K. (2010). Systemic melioidosis presenting as suppurative parotitis. Indian Pediatr..

[159] Gauthier J., Gérôme P., Defez M., Neulat-Ripoll F., Foucher B., Vitry T., Crevon L., Valade E., Thibault F.M., Biot F.V. (2016). Melioidosis in Travelers Returning from Vietnam to France. Emerg. Infect. Dis..

[160] Loh T.L., Latis S., Crossland G., Patel H. (2017). Disseminated melioidosis in the head and neck. BMJ Case Rep..

[161] Laowansiri P., Shah S., Kiratisin P., Apisarnthanarak A. (2009). A role for carbapenem in the treatment of melioidosis in developing countries?. Int. J. Infect. Dis..

[162] Wijekoon S., Prasath T., Corea E.M., Elwitigala J.P. (2014). Melioidosis presenting as lymphadenitis: A case report. BMC Res. Notes.

[163] Brent A.J., Matthews P.C., Dance D.A., Pitt T.L., Handy R. (2007). Misdiagnosing melioidosis. Emerg. Infect. Dis..

[164] Salam A.P., Khan N., Malnick H., Kenna D.T., Dance D.A., Klein J.L. (2011). Melioidosis acquired by traveler to Nigeria. Emerg. Infect. Dis..

[165] Baker K., Duncan T., Kung S., Smith S., Hanson J. (2024). Melioidosis masquerading as malignancy in tropical Australia; lessons for clinicians and implications for clinical management. Acta Trop..

[166] Thanathanee O., Charoensuk K., Uthairat Y., Laohapitakvorn S., Anutarapongpan O., Suwan-Apichon O. (2023). Case Report: *Burkholderia pseudomallei*-Caused Sclerokeratitis. Am. J. Trop. Med. Hyg..

[167] Chen K.J., Sun M.H., Hou C.H., Sun C.C., Chen T.L. (2007). *Burkholderia pseudomallei* endophthalmitis. J. Clin. Microbiol..

[168] Gee J.E., Bower W.A., Kunkel A., Petras J., Gettings J., Bye M., Firestone M., Elrod M.G., Liu L., Blaney D.D. (2022). Multistate Outbreak of Melioidosis Associated with Imported Aromatherapy Spray. N. Engl. J. Med..

